# Spaced cognitive training promotes training transfer

**DOI:** 10.3389/fnhum.2014.00217

**Published:** 2014-04-10

**Authors:** Zuowei Wang, Renlai Zhou, Priti Shah

**Affiliations:** ^1^Department of Psychology, Combined Program in Education and Psychology, University of MichiganAnn Arbor, MI, USA; ^2^Beijing Key Laboratory of Experimental Psychology, School of Psychology, Beijing Normal UniversityBeijing, China; ^3^State Key Laboratory of Cognitive Neuroscience and Learning, Beijing Normal UniversityBeijing, China

**Keywords:** cognitive training, working memory, transfer, schedule, spacing, primary school, children, fluid intelligence

## Abstract

Cognitive training studies yield wildly inconsistent results. One dimension on which studies vary is the scheduling of training sessions (Morrison and Chein, 2011). In this study, we systematically address whether or not spacing of practice influences training and transfer. We randomly assigned 115 fifth grade children to an active control group or one of four training groups who received working memory training based on a “running span” task (Zhao et al., 2011). All groups received the same total amount of training: 20 sessions of training with 60 trials for an average of 20 min per session. The training was spread across 2, 5, 10, or 20 days. The active control group received 20-min sessions of math instruction for 20 sessions. Before and after training participants in all five groups performed a single transfer test that assessed fluid intelligence, the Raven's Progressive Matrices Test. Overall, participants in all four training groups improved significantly on the training task (at least partially), as reflected by increased speed. More importantly, the only training group to show significant improvement on the Raven's was the group who had the greatest amount of spacing (20 days group) during training and improvement in this group was significantly higher than that of the control group.

## Introduction

Working memory is the cognitive system that actively maintains and processes information for human problem solving (Miyake and Shah, 1999). Working memory training has been extensively studied in recent years (e.g., Klingberg, 2010; Morrison and Chein, 2011). Studies have shown that working memory training is beneficial to various subject populations, varying from young children to old adults, including healthy subjects as well as those with special needs. For example, working memory training has been found to reduce the symptoms of ADHD children (Klingberg et al., 2002, 2005), facilitate the recovery of cognitive functions in patients after stroke (Westerberg et al., 2007), enhance “old-old” adults' memory performance (80 years old; Buschkuehl et al., 2008), and improve fluid intelligence in pupils (Jaeggi et al., 2011; Zhao et al., 2011) as well as college students (Jaeggi et al., 2008, 2014).

The significance of working memory training is largely dependent on the potential transfer effects to other untrained situations. Due to the various transfer effects identified by previous studies, some researchers view working memory training as promising for general cognition enhancement (see the review by Morrison and Chein, 2011). However, while researchers almost always find performance improvement on trained tasks, not all studies find transfer. In fact, some researchers argue that there is little to no solid evidence that true far transfer effects arise from working memory training (Shipstead et al., 2012; Melby-Lervåg and Hulme, 2013; Rapport et al., 2013; Redick et al., 2013).

Efforts have been taken to investigate factors that affect successful training and transfer. Three broad classes of factors are likely relevant: individual characteristics of participants receiving training, the nature of the training task, and conditions of training. Individual characteristics that affect training and transfer may include initial ability of participants, the underlying source of any deficits in working memory performance, and motivational factors. Research in our laboratory has found, for example, that individuals who believe that intelligence is a malleable construct are more likely to benefit from training than those who believe intelligence is fixed (Jaeggi et al., 2014). Individuals who report that a training task is “too difficult” seem to disengage and show less improvement than individuals who enjoy the challenge of a task (Jaeggi et al., 2011). Other individual motivational factors may include the degree to which individuals are intrinsically motivated (Katz et al., in press; Shah et al., 2012).

The nature of the training task(s), of course, will also influence what types of transfer might be found. In a classic review of training and transfer, Schmidt and Bjork (1992) emphasize the importance of using a variety of tasks to increase the likelihood that training effects are not task-specific. Likewise, tasks that are adaptive and cognitively challenging may also limit the extent to which task-specific strategies that can be developed. In the original Jaeggi et al. (2008) study, the use of the dual n-back task that required maintaining and updating information in both visual and auditory modalities may have limited the ability of individuals to develop specific rehearsal strategies. More generally, Morrison and Chein (2011) found in their review of working memory training that studies that focus on training strategies were not as effective as those that focused on core working memory capacity. Some characteristics of the training task may also interact with motivation or perceived difficulty of a task. We found, for example, that in a brief, 3-day cognitive training intervention that some game-like features may in fact serve as a distraction making cognitive training more difficult (Katz et al., in press, this issue).

A third class of factors that affect the effectiveness of cognitive training is the dosage or sheer amount of training (Jaeggi et al., 2008) as well as the training schedule (Schmidt and Bjork, 1992). It has been known for over 100 years that individuals remember information better under spaced practice conditions compared to massed practice (Ebbinghaus, 1885). This spacing effect has been extensively studied under numerous contexts since then. Most of these studies showed that spaced learning led to better learning outcomes (for a review, see Cepeda et al., 2006). For example, spaced learning is beneficial to foreign word learning (Bahrick et al., 1993), inductive learning (Kornell and Bjork, 2008; Vlach et al., 2008), and professional training (Hagman, 1980). The spacing effect is also found in cognitive skill acquisition tasks for both human and animal subjects (Shebilske et al., 1994, 1999; Sisti et al., 2007). For example, Sisti et al. (2007) trained rats using different schedules on a water maze task and they found that the rats that received more spaced training outperformed rats who received massed training. More interestingly, performance was correlated with the survival of new neurons in the hippocampus, suggesting that spaced training elicited more neural changes.

Shebilske et al. (1999) investigated the spacing effect in a complex skill acquisition task that is perhaps closest to the types of cognitive training tasks of interest in the current paper. The training task they used was Space Fortress, a task that required “short- and long-term memory loading, high workload demands, dynamic attention allocation, decision making, prioritization, resource management, continuous motor control, and discrete motor responses.” In their study, college students received training on this task for a total of 10 h with two different schedules, either within 10 days or within 2 days. Results showed that the more spaced training group had an advantage in the acquisition, retention and training transfer to a different device (from joystick to keyboard). In addition, the more spaced training group also showed less interference when asked to perform the Space Fortress task together with a secondary tapping task. The spacing effect in skill acquisition is also studied in the context of online gaming. Stafford and Dewar (2014) analyzed gameplay data of 854,064 players. They found that players who spread their practice tended to achieve higher score in the game.

There are many theoretical explanations for the spacing effect, most of which are not mutually exclusive. Spaced learning is consistent with rational models of memory that assume memory is adaptive (Anderson and Schooler, 1991). Exposure to information in a spaced manner is a clue to the memory system that the information may be needed again at a future date. By contrast, massed practice may support storing information for the short term, as the information is not again needed after a short period of practice. More specific theoretical explanations for spacing effects in memory have also been proposed, including the “deficient-processing hypothesis” (Greene, 1989), the “context variability” hypothesis (Greene, 1989), the incubation effect (for a review, see Sio and Ormerod, 2009), sleep-dependent memory consolidation (Stickgold, 2005) and so on. These theoretical explanations are likely to hold not only in the context of general skill acquisition and memory, but also specifically in the context of cognitive training. The deficient-processing theory, for example, posits that when too much information is presented to participants in memory tasks, the information is processed with lower efficiency. The same rationale could also be applied to working memory training. Thus, massed training may not elicit the neural changes that are necessary for the training transfer.

In summary, the spacing effect in memory may shed light on the understanding of similar effects in cognitive training. However, cognitive training also has some unique features. The increase in the capacity and speed of cognitive processing cannot be treated similarly as the acquisition of new knowledge. For example, the spacing effect in cognitive training may also show different patterns than that in memory: the spacing effect in memory tasks may be a result of more covert rehearsal, whereas in skill acquisition (such as motor behavior), it is likely to be related to “effort, work, reactive inhibition, or fatigue” (Adams, 1987). In memory tasks the optimal spacing “gap” is greater when the delay between practice and final test is longer (Cepeda et al., 2008). However, the optimal spacing in skill acquisition situations still remains unknown (Stafford and Dewar, 2014).

Currently, we are not aware that any working memory training studies have systematically varied the schedule under which individuals are trained to investigate the effect of training on outcome. A potential spacing effect in working memory training has both theoretical significance and important practical implications. Theoretically, a systematic investigation of the spacing effect in working memory training may help clarify the mixed findings in the current working memory training literature. Studies have revealed different effect size in training gains and training transfers, which could be a result of uncontrolled training schedule. In practice, an optimized training schedule may produce stronger and broader training gains in a shorter time, which cuts the training cost and allows more people to benefit from it.

In the current study, we investigated the effect of different training schedules on the outcome of working memory training in 5th grade classes in Muling, China. We used the same intervention that was originally used in Zhao et al. (2011). In that study, the intervention was a running span task in which 4th grade children from China were presented with a sequence of either animal drawings or locations on a 3-by-3 grid. The task required them to recall the three most recent stimuli in the presented order when the sequence stopped. Using a pre-test, training, post-test paradigm, they found that 20 sessions of training significantly improved children's performance on the Raven's Standardized Progressive Matrices (SPM). In this study we used the same running span training task used by Zhao et al. (2011) for several reasons: (1) this updating task has already been shown to improve fluid reasoning in one study, (2) the task had already been used with children in China and was designed to be appealing and engaging to children of the same range with a similar cultural background. Our study differed from the Zhao et al. (2011) study in that we included an active control group that was educational in content (extra math instruction with their teachers). All participants in the training groups received the identical amount of practice on the training task (20 total sessions, an amount of training that has been used in other training studies that found transfer of training such as Zhao et al., 2011). However, the groups differed in the spacing of the training sessions. One group of participants received all 20 sessions within 2 days (10 sessions per day), the second group received all 20 sessions within 5 days (four sessions per day), the third group received the training within 10 days (two sessions per day), and the fourth group, with the greatest spacing, received one session of training per day for 20 days.

Based on the body of research on spacing, memory, and skill acquisition, we predicted that training schedule would have a substantial impact on working memory training gain and transfer. Specifically, we predicted that the group(s) with the most spacing of training would improve most on the training task and furthermore show the most transfer. In addition to this primary goal, we wished to replicate the results of other studies that have trained memory updating and found transfer to fluid intelligence in children (Jaeggi et al., 2011; Zhao et al., 2011). The total number of studies in which updating is trained in typically developing children is rather small, and thus this study provides an additional data point with respect to the potential effects of updating training more generally.

## Materials and methods

### Participants

A total of 115 5th grade students (10–11 years old) from Muling Shiyan Elementary School (Muling, China) were recruited to participate in the study. Before the training, they were told that upon finishing the training they would receive different gifts based on their performance in the training, including school bags, fountain pens and lockable notebooks. Twenty subjects were unable to strictly follow their assigned training schedule, or were absent during the pre-test or post-test thus dropped out from the study, resulting of 95 valid subjects in the data analysis (52 female). There was no group difference on the dropout rate, ×^2^ (4, *n* = 115) = 3.31, *p* = 0.51. Due to computer error, two subjects' Grid task training data were lost, and one subject's Animal task training data were lost.

### Design

Participants were randomly assigned into one of the four training groups or an active control group. All the four training groups received the same total amount of training: 20 sessions of training with 60 trials for an average of 20 min per session. The training was spread across 2, 5, 10, or 20 days. The control group stayed with their teachers in their classrooms (after school) for 20 min each day for 20 days and received instruction focused primarily on math. The gender distribution in the five groups was: 20 Days—9 female 11 male; 10 Days—11 female 9 male; 5 Days—12 female 8 male; 2 Days—10 female 5 male; control group—10 female 10 male.

Before and after training, participants were all tested on a measure of fluid intelligence, the Raven's Standard Progressive matrices test. We compared pre-test to post-test improvements in the five groups (four training and one control) to assess transfer.

### Materials and procedure

#### Training task

We used two forms of the “running span” task for the training (Zhao et al., 2011). In one task (Animal), subjects saw pictures of different animals presented sequentially on the computer screen. In the other task (Grid), subjects saw a cartoon figure moving sequentially to different locations in a 3 × 3 grid. In both of the tasks, the length of the sequence randomly varied from 5, 7, 9, or 11 items in a row and participants did not know ahead of time how many items would be presented prior to recall instructions. At the end of each sequence of trials, subjects were asked to recall the three most recent stimuli in the presented order. Because a subject could not predict when the sequence would end, they were required to continuously update their working memory with the most recent three items.

Each session consisted of participants performing one set of animal and one set of grid trials. Each set consisted of 30 trial sequences that were divided into six blocks of five trials each. Within each block, if subjects provided the correct response for three or more trials, the presentation time of each stimulus in the next block would decrease by 100 ms, thus making the task more difficult. If participants got fewer than three trials correct, the presentation time of each stimulus would increase by 100 ms, which made the next block easier. For both the Animal and the Grid tasks, the starting presentation time was 850 ms for the 20, 5, and 2 days groups. The starting presentation time for the 10 days group was about 1000 ms for the Animal task and 950 ms for the Grid task (this was due to a computer error: the recovery mode of the training computers wasn't turned off and the 10 days group started from where the 20 days group left after their first session).

We decided that subjects' performance on the training tasks could be reflected by the presentation time of the stimulus. To track their training performance, we calculated the averaged presentation time for each session (both Animal and Grid), and named this measure as “presentation time” for that set of the session hereafter. To encourage children to try their best on the training tasks, correct response on each trial would earn them one point, which was shown by adding one smiley face to a feedback chart which was located at the bottom of the screen. The total points (smiley faces) could be used to trade for different gifts (school bags, fountain pens and lockable notebooks) after the training. More detailed descriptions of the training tasks can be found in Zhao et al. (2011).

#### Math instruction for control group

When the 20 days training group received the daily training, the active control group remained in their classroom and worked with their math teachers. They received extra math exercises from a 5th grade mathematics workbook for 20 min every day. Students first worked on problems from the workbook, and then the teacher checked their answers and provided further instruction when necessary. Some example problems the students practiced include: solving equations with one variable, calculating the area of different shapes that required them to divide the shapes into regular shapes with known formulas for area calculation, word problems (e.g., calculating the distance of moving objects, sometimes requiring the use of equations) etc. No rewards were provided for the control group.

#### Transfer task

The Raven's Standard Progressive Matrices (SPM) was used to evaluate the transfer effect of the training, following the design of a number of working memory training studies (e.g., Jaeggi et al., 2008; Zhao et al., 2011). Specifically, the 60 items in SPM were split into two subtests: odd numbered items and even numbered items, which were used in pre-test and post-test with counterbalance. We also used the TONI (Test of Nonverbal Intelligence; Brown et al., 2010) for some participants, but due to scheduling difficulties we were not able to collect TONI test scores for the 20 session training or active control group. Therefore, only Raven's scores are used in the analyses.

### Procedure

All children in the training groups were given one half of the SPM (even or odd items) as a pre-test before they started the training. Whether they received odd or even items at pre-test was counterbalanced. Children in all the groups were given the pre-test within the same 3 days prior to the training on the 20 days group. Thus, the distance between pre-test and post-test for all groups was approximately the same.

During the training, each training session consisted of one set of Animal task and one set of Grid task. Children in the 20 days group received one session every day after school, which took about 15–25 min. Children in the 10 days group received one session during the 2-h-long noon recess (for 15–25 min) and another session after school (another 15–25 min). Children in the 5 days group received two sessions during the noon recess (i.e., 30–50 min) and another two sessions after school (an additional 30–50 min). Children in the 2 days group received the training after the semester, and they finished the 20 total sessions within 2 days (approximately 10 sessions per day for a total of 150–250 min each day with rest and lunch breaks). For the 5 Days and 2 Days group, children were given a 5–10 min rest after approximately every 30–40 min of training. In all the groups, the very first training session was used as a practice session in which children were allowed to stop and ask questions. Thus, training data for the first session was not recorded and not included in the analysis.

After the training, children were given the alternate version of the SPM as a post-test. For the 5 and 2 days group, the SPM was administered the day following training completion to prevent decreased performance due to training fatigue. We were mindful about keeping the time interval between the pre-test and post-test the same for all the five groups. However, due to scheduling difficulties this interval for the 5 and 2 Days group was about 1 week longer.

Children in all the groups strictly adhered to their training schedule. Before weekends or holidays, we made arrangements with all the parents to make sure their children would come to school for the training. Three children in the 20 Days group and 2 in the 10 Days group who lived too far to get to the school received the training at home with the experimenter overseeing their training using remote desktop.

Children in none of the groups were given any information about how the Raven's pre- and post-tests may be related to training/math learning. Before working on the Raven's tests, they were just told that they were to work on some puzzles. Children in all the four training groups were motivated to earn more points by correctly recalling animals/grid locations during the training for better reward; children in the control group were motivated to learn math because they were to receive a math test after the 20 days.

The study was reviewed and approved by the Institutional Review Board at the University of Michigan. Informed consent was obtained from all the parents whose children participated in the study. Before each training/testing session, oral assent was also obtained from all the children who participated.

## Results

### Training gain

**Figure 1:**
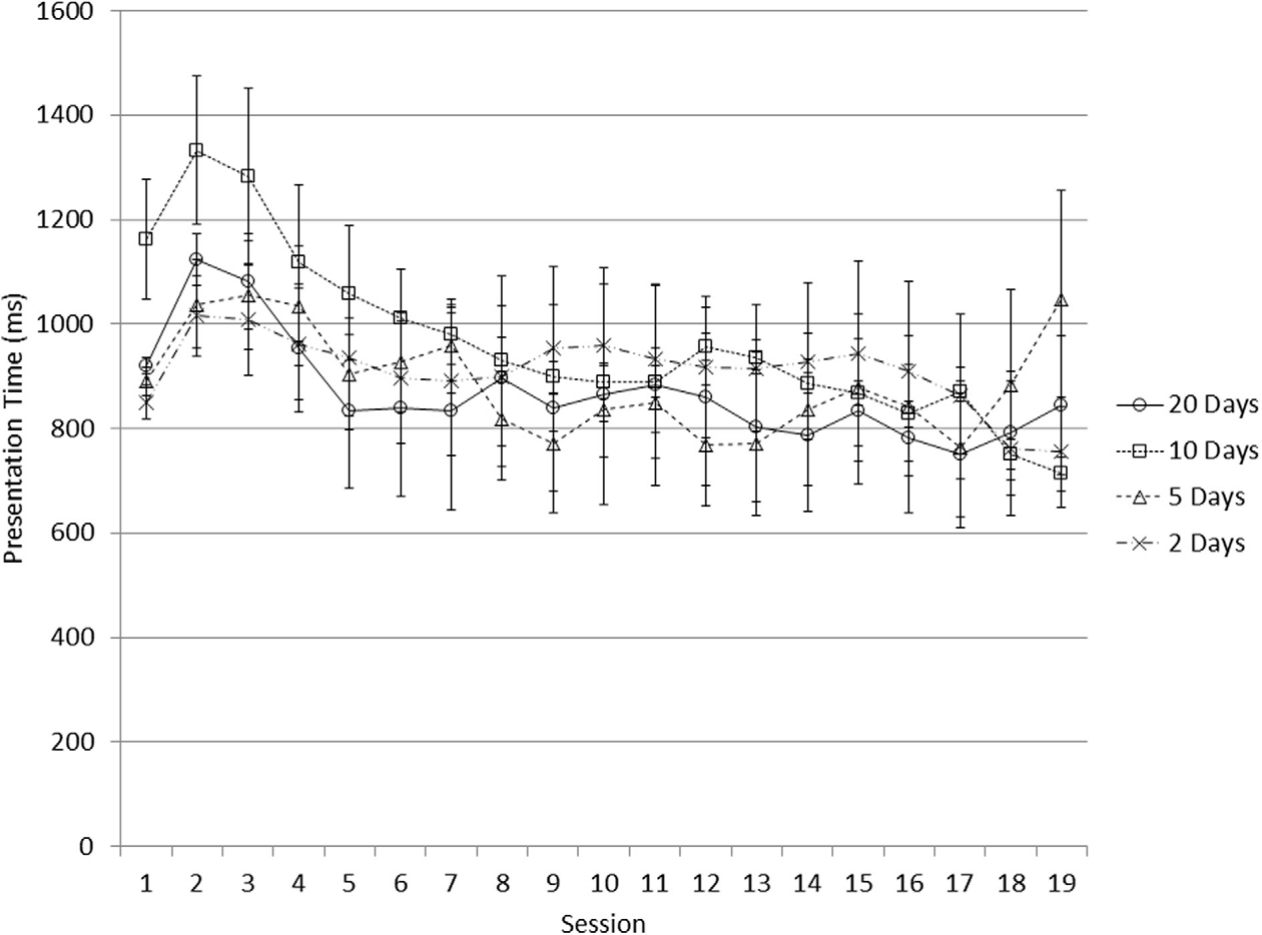
**Presentation time decreased with training on the Animal task; error bars represent standard error**.

**Figure 2:**
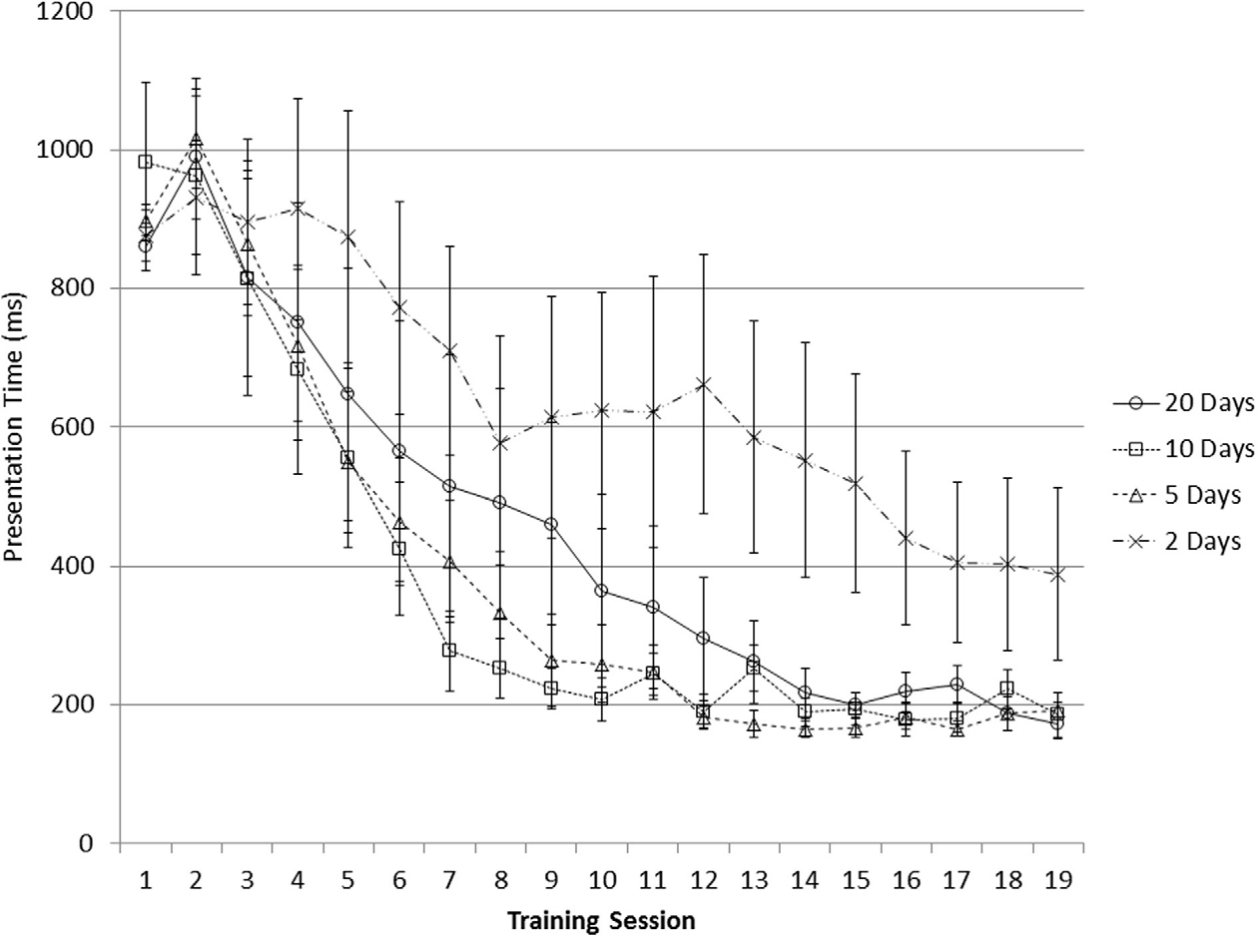
**Presentation time decreased with training on the Grid task; error bars represent standard error**.

The five groups had similar scores in the Raven's pre-test, *F*_(4, 90)_ = 0.20, *p* = 0.938. Training performance is summarized in Figures 1, 2. Improvement on the two running span tasks is reflected by subjects' increasing capability to process faster presentation of the stimuli (i.e., increased speed). To evaluate the training gain, we subtracted the averaged presentation time of the first three sessions from that of the last three sessions, which represented the processing speed increase as a result of the training (here negative values indicate improvement). Based on this measure, all the four training groups showed significant improvement on the Grid task; both the 20 Days group and the 10 Days group made significant progress on the Animal task (one-sample *t*-test compared to the value “0”; Table 1). However, a comparison among the four groups did not show significant difference in training gain among the groups in either the Animal task, *F*_(3, 70)_ = 1.288, *p* = 0.285, nor the Grid task, *F*_(3, 69)_ = 1.077, *p* = 0.365.

**Table 1:** **One-sample *t*-test showing training gain of the four training groups as measured by the presentation time decrease (the average of the last three sessions minus that of the first three sessions)**.

	**Animal task**				**Grid task**			
	**RT (ms) decrease**	***t***	***p***	***df***	**RT (ms) decrease**	***t***	***p***	***df***
20 Days group	−246	−2.30	0.033	19	−691	−8.69	<0.001	19
10 Days group	−481	−3.57	0.002	18	−722	−5.12	<0.001	18
5 Days group	−124	−0.675	0.507	19	−743	−11.9	<0.001	19
2 Days group	−165	−1.54	0.147	14	−502	−4.62	<0.001	13

^*^Note: training data for one subject in the 10 Days group (both Animal and Grid) and one subject in the 2 Days group (Grid) were lost due to computer error; the starting stimulus presentation time for the 10 days group was longer than the other groups (~150 ms longer in the Animal task and ~100 ms longer in the Grid task) thus resulting in a relatively larger training gain in the Animal task, but not in the Grid task. See Figures 1, 2 for details.

Training gain can also be measured by the regression slope of presentation time (defined in Training task) on session number as an indication of the session-by-session processing speed improvement. In both Figures 1, 2, the presentation time was the average presentation time of the six blocks within a session. From these figures, subjects' presentation time first showed a slight increase then a steady decrease, suggesting the starting presentation time was appropriate. Table 2 shows a summary of these regression slopes for the four training groups on the two running span tasks. There was no significant difference in training gain (reflected by the regression slope) among the four training groups in either the Animal task, *F*_(3, 70)_ = 0.913, *p* = 0.439, or the Grid task, *F*_(3, 69)_ = 0.534, *p* = 0.660.

**Table 2:** **Regression slopes reflecting averaged session-wise stimulus presentation time decrease (in milliseconds; standard errors of the slopes provided in the parenthesis)**.

	**Animal task**	**Grid task**
20 Days Group	−74(32)	−261(47)
10 Days Group	−156(47)	−247(50)
5 Days Group	−43(75)	−265(32)
2 Days Group	−41 (43)	−187(54)

It should be noted that subjects' accuracy was also tracked. However, due to the nature of the task, subjects' accuracy only improved during the first few sessions and then remained stable. This is because if their accuracy in a given block (five trials), the presentation speed would become faster in the next block.

### Training transfer

**Figure 3:**
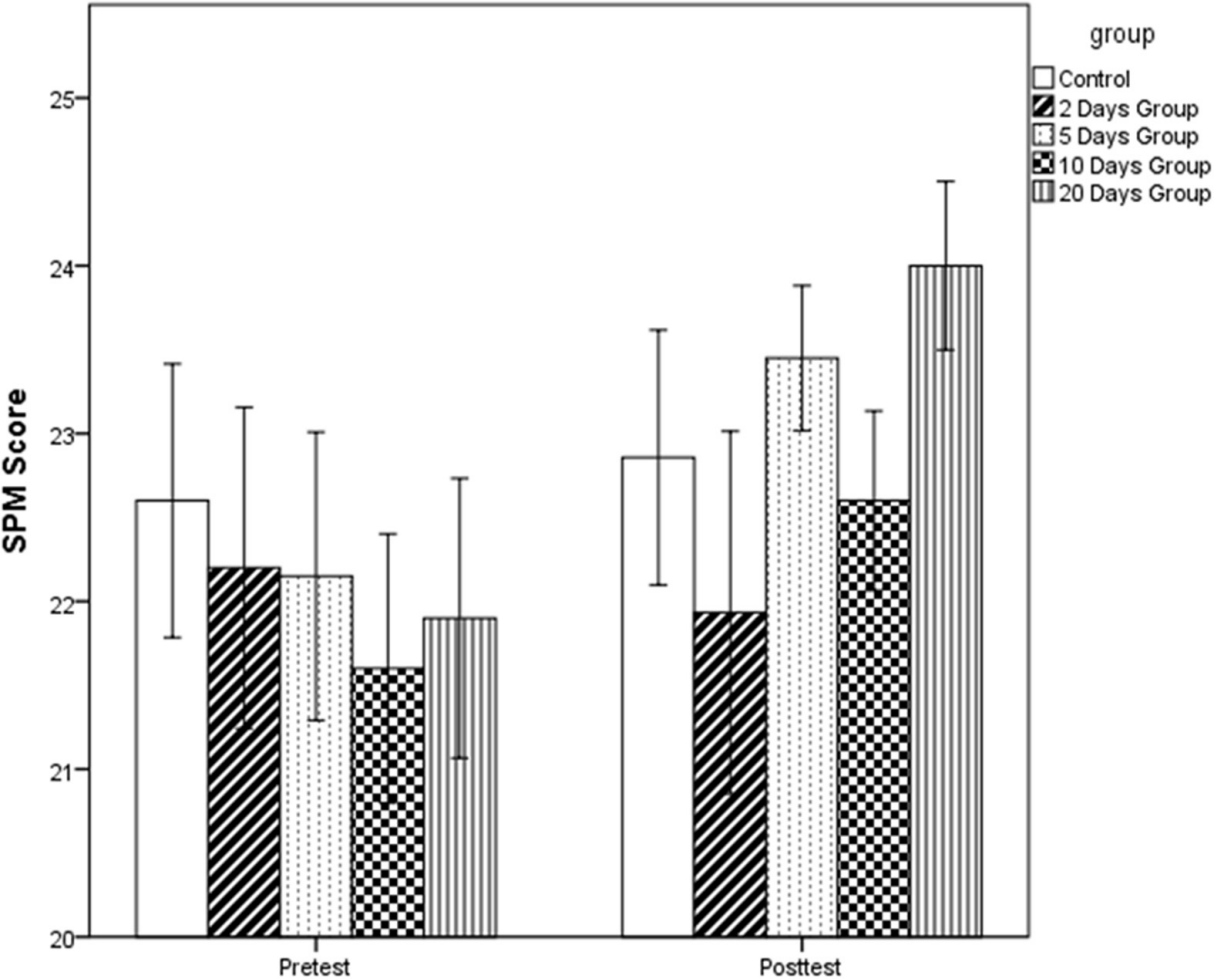
**Scores on the SPM before and after training; error bars represent standard error**.

A Paired-sample *t*-test was performed for each of the four training groups together with the control group on SPM pre-test and post-test to evaluate the training transfer. Results show that only the 20 Days group showed significant improvement on the test (Table 3; for original score, see Figure 3). Because only the 20 Days training group showed evidence of improvement on the transfer task, we compared gain on the SPM (post-test minus pre-test scores) for the 20 Days group and the active control group. The SPM gain in the 20 Days group was significantly larger than the control group, *t*_(38)_ = 1.832, *p* = 0.038 (one-tail test), Cohen's *d* = 0.59.

**Table 3:** **Improvement on SPM after the training as reflected by paired-sample *t*-test**.

	***t***	***df***	***p***	**Cohen's *d***
20 Days group	2.93	19	0.009	1.34
10 Days group	1.27	19	0.220	0.58
5 Days group	0.95	19	0.355	0.44
2 Days group	0.19	14	0.854	0.10
0 Days group (Control)	0.19	19	0.855	0.09

Table 3 also shows that the effect size (Cohen's *d*) decreases as the students received a more massed training schedule. Thus, we conducted a regression analysis to see whether training schedule had an effect on the SPM post-test after controlling for pre-test scores. In the regression analysis, training schedule was entered as the number of days completing the training (i.e., 20, 10, 5, or 2), with the control group a value of “0.” Results show that Training Schedule had an effect on the post-test score after controlling for pre-test score, *p* = 0.052 (2-tailed).

### The relationship between training gain and transfer

To provide further support for the idea that practice on the running span task was directly related to improvement on the transfer measure of fluid intelligence, the SPM, we assessed the relationship between improvement on the training task and the magnitude of the transfer effect. Each subjects' training gain is measured by the averaged session-by-session presentation time decrease (i.e., processing speed increase), which is calculated as the regression slope of each subject's presentation time by the session number. The magnitude of the transfer effect is the score difference between SPM post-test and pre-test. Table 5 summarizes the correlation coefficients of training gain and training transfer. It can be seen that only for the most spaced training schedule does the training gain significantly correlate with transfer, *r*_(20)_ = 0.465, *p* < 0.05.

## Discussion

This study assessed the effect of spacing of working memory training on the Raven's SPM. We predicted that spacing of training would affect both training gains as well as transfer, but found that there were no significant differences in training gain (as measured by reaction time) across the groups. Importantly, however, we did find a significant effect of training schedule on *transfer*. Only participants in the most spaced group, 20 sessions with one session per day, showed significant improvement on the Raven's SPM. Furthermore, improvement on SPM test performance for this group was significantly different from that of the active control group. More generally, there was a significant relationship between training schedule and transfer such that the more spaced the training, the greater the transfer (see Table 4).

**Table 4:** **Regression analysis showing the effect of training schedule on SPM post-test**.

	***B***	***SEB***	β	***p***
**Model 1**				
Pre-test	0.279	0.078	0.349	<0.001
**Model 2**				
Pre-test	0.289	0.077	0.361	<0.001
Training schedule	0.076	0.038	0.189	=0.052

**Table 5:** **Correlation between training gain (RT decrease) and magnitude of training transfer**.

**Score change in SPM**	**Averaged training gain**
20 Days group (*n* = 20)	−0.465^*^
10 Days group (*n* = 19^**^)	+0.046
5 Days group (*n* = 20)	−0.015
2 Days group (*n* = 14^**^)	−0.135

* p < 0.05;

** *training gain data were lost from one subject in the 10 Days group and one subject from in 2 Days group so the n's here do not match the df's in Table 3*.

We can draw two main conclusions from this study. First, training schedule has a significant impact on transfer of training. Second, the transfer effect of the 20-day group replicated results of a recent study that used the identical training and transfer tasks (Zhao et al., 2011). It is also consistent with studies that have found improvements in typically developing children following working memory training (Thorell et al., 2009; Jaeggi et al., 2011; Zhao et al., 2011).

One question that arises is why this study and some others find far transfer effects whereas others do not. As discussed above, our study had several features associated with successful training studies: we tested children and not adults (Morrison and Chein, 2011), we used an adaptive training task that increased as performance increased (Jaeggi et al., 2008), we carefully monitored children's training schedules, children were given token extrinsic rewards not only for participation but also performance, multiple training tasks were used to ensure task variability and reduce the likelihood that children develop task-specific strategies (Schmidt and Bjork, 1992), and the working memory updating task taxed both active maintenance and interference resolution processes (Jaeggi et al., 2011). Further, the students who participated in this study appeared to be highly motivated; while the drop-out rate of many cognitive training studies is relatively high, 95 out of the initially recruited 115 completed the entire study.

Interestingly, our study found that training task improvements, at least in the group that received training across 20 days, was correlated with improvement in the transfer task. This result is consistent with the Jaeggi et al. (2011) study that found transfer only for participants who made significant improvements on the training task. Moreover, our study extends the picture. Training gain is not solely predictive of transfer outcome—participants who received the massed training schedules also improved on the training task the same extent as those who were in the most spaced group, but they did not show significant improvement on the transfer task. Thus, the relationship between training and transfer was significant only for the most spaced group. It is possible that individuals in the massed groups learned more task-specific strategies whereas those in the spaced group were able to focus on the underlying cognitive skills. This explanation supports the deficient-processing hypothesis (Greene, 1989), which assumes that when too much information presented in a short period of time, it is processed with lower efficiency. In our working memory training context, this means that training that happens within a massed schedule does not induce deep processing that may contribute to training transfer. However, it is not entirely clear what exactly explains the dissociation between improvement and transfer for the massed groups. Future research should address the degree to which spacing might affect strategy use.

As the first study that we are aware of that explores the spacing effect in working memory training, the current study has some limitations that need to be addressed by future research. First, the four different training schedules we used did not fully represent the schedule variation of the current training studies. According to Morrison and Chein (2011), the schedule of current working memory training studies varied from 2 to 14 weeks. In the current study we only tested the lower end of this continuum. It is yet to be explored the magnitude of the transfer when the training is spaced over a longer period of time. Second, due to the small sample size, we used relatively less conservative statistical tests, including running planned contrast without adjustment of α level (we directly compared the 20 Days group with the control group using a one-tailed test with α = 0.05). Based on the effect size of this study, we suggest future studies use a sample size twice as large as ours (i.e., 40 subjects per training schedule). Third, because we only used one training task and one transfer task, the reliability of this spacing effect in working memory training is limited to these specific tasks. In addition, the use of a single far transfer task did not allow us to evaluate the mechanism of the transfer effect. Thus, replicating these findings with a larger battery of transfer tasks including both near and far measures is an important next step.

In conclusion, this study demonstrated that training schedule has substantial impact on transfer of training. More research that investigates the moderators of training may help shed light on the debate of whether working memory training leads to broader cognitive improvement.

### Conflict of interest statement

The authors declare that the research was conducted in the absence of any commercial or financial relationships that could be construed as a potential conflict of interest.
